# Deregulation of secondary metabolism in a histone deacetylase mutant of *Penicillium chrysogenum*

**DOI:** 10.1002/mbo3.598

**Published:** 2018-03-24

**Authors:** Fernando Guzman‐Chavez, Oleksandr Salo, Marta Samol, Marco Ries, Jeroen Kuipers, Roel A. L. Bovenberg, Rob J. Vreeken, Arnold J. M. Driessen

**Affiliations:** ^1^ Molecular Microbiology Groningen Biomolecular Sciences and Biotechnology Institute University of Groningen Groningen The Netherlands; ^2^ Kluyver Centre for Genomics of Industrial Fermentations Delft The Netherlands; ^3^ Division of Analytical Biosciences Leiden/Amsterdam Center for Drug Research Leiden The Netherlands; ^4^ Netherlands Metabolomics Centre Leiden University Leiden The Netherlands; ^5^ Department of Cell biology University Medical Center Groningen Groningen The Netherlands; ^6^ Synthetic Biology and Cell Engineering Groningen Biomolecular Sciences and Biotechnology Institute University of Groningen Groningen The Netherlands; ^7^ DSM Biotechnology Center Delft The Netherlands; ^8^ Present address: Rob J. Vreeken, Discovery Sciences Janssen R &D Beerse Belgium

**Keywords:** chrysogine, crosstalk, histone deacetylase, naphtha‐γ‐pyrone, *Penicillium chrysogenum*, sorbicillinoids

## Abstract

The *Pc21 g14570* gene of *Penicillium chrysogenum* encodes an ortholog of a class 2 histone deacetylase termed HdaA which may play a role in epigenetic regulation of secondary metabolism. Deletion of the *hdaA* gene induces a significant pleiotropic effect on the expression of a set of polyketide synthase (PKS) and nonribosomal peptide synthetase (NRPS)‐encoding genes. The deletion mutant exhibits a decreased conidial pigmentation that is related to a reduced expression of the PKS gene *Pc21 g16000* (*pks17*) responsible for the production of the pigment precursor naphtha‐γ‐pyrone. Moreover, the *hdaA* deletion caused decreased levels of the yellow pigment chrysogine that is associated with the downregulation of the NRPS‐encoding gene *Pc21 g12630* and associated biosynthetic gene cluster. In contrast, transcriptional activation of the sorbicillinoids biosynthetic gene cluster occurred concomitantly with the overproduction of associated compounds . A new compound was detected in the deletion strain that was observed only under conditions of sorbicillinoids production, suggesting crosstalk between biosynthetic gene clusters. Our present results show that an epigenomic approach can be successfully applied for the activation of secondary metabolism in industrial strains of *P. chrysogenum*.

## 1 INTRODUCTION

During the last decades, the filamentous fungus *Penicillium chrysogenum* has been used extensively in industry for the production of the β‐lactam antibiotic penicillin (Fleming, 1929). The biosynthetic pathway and the corresponding genes involved have been well described and current production strains are generated for the high‐level production of penicillins through the implementation of an intense classical strain improvement program. However, the full potential of secondary metabolism of *P. chrysogenum* largely remained unknown till the genomic sequence became available (Van Den Berg et al., 2008). The genome specifies multiple genes for secondary metabolite formation including 20 polyketide synthases (PKSs), 10 nonribosomal peptide synthetase (NRPSs), 2 hybrids (PKS‐NRPS), and 1 dimethylallyltryptophan synthase. The function of most of these genes remains unknown (Samol, Salo, Lankhorst, Bovenberg & Driessen, 2016; Van Den Berg et al., 2008). Recently, a genome‐based identification and analysis of the roquefortine meleagrin NRPS gene cluster was performed for *P. chrysogenum* (Ali et al., 2013; Garcia‐Estrada et al., 2011; Shang, Zhang, & Zheng, 2013; Veiga et al., 2012). However, unlike the roquefortine gene cluster, the expression level of the majority of the secondary metabolite genes under laboratory conditions is low (Van Den Berg et al., 2008). Therefore, more elaborate methods other than gene inactivation are required for identification and further analysis of these so‐called “silent” secondary metabolite genes.

New approaches have evolved during the postgenomic era to activate gene clusters such as interference with cluster specific regulatory genes or even of pleiotropic regulator of chromatin structure like LaeA. This has triggered the research on the cryptic potential of fungal secondary metabolism (Brakhage & Schroeckh, 2011). A potential powerful approach is the epigenetic regulation of gene expression. In eukaryotic cells, DNA is compacted into a complex chromatin structure. The histone proteins H2A, H2B, H3, and H4 form the core histone octamer complex with DNA called nucleosome, the structural and functional unit of the chromatin (Luger, 2003). The formation of the nucleosomes may interfere with the recognition of the bound DNA by various transcriptional elements causing gene silencing (Lee, Hayes, Pruss, & Wolffe, 1993). Thus, remodeling of the chromatin by the histone modifications is a trigger that influences transcription, replication, and DNA repair (Yu, Teng, Waters, & Reed, 2011; Zhu et al., 2011). The histone acetylation status is controlled by the balanced activity of histone acetylases (HATs) and deacetylases (HDACs) (Brosch, Loidl, & Graessle, 2008). Hyperacetylation of the chromatin induced by deletion or chemical inhibition of HDACs leads to euchromatin formation and transcriptional activation of silent chromosomal regions (Gacek & Strauss, 2012). Cladochromes and calphostin B in *Cladosporium cladosporioides* and nygerone A from *Aspergillus niger* are secondary metabolites that have recently been identified with this strategy using the HDAC inhibitor suberoylanilide hydroxamic acid (SAHA) (Carafa, Miceli, Altucci, & Nebbioso, 2013; Fisch et al., 2009; Henrikson, Hoover, Joyner, & Cichewicz, 2009). An altered secondary metabolite profile was also reported for *Alternaria alternata* and *Penicillium expansum* treated with HDAC inhibitor Trichostatin A (TSA) (Shwab et al., 2007).

Histone deacetylases are represented by two protein families: the ‘‘classical’’ HDACs and the recently established group of NAD^+^‐dependent sirtuins (de Ruijter, van Gennip, Caron, Kemp, & van Kuilenburg, 2003). Members of both families were initially described in *S. cerevisiae* and subsequently identified in filamentous fungi and humans (Taunton, Hassig, & Schreiber, 1996). The ortholog of the RPD3 (reduced potassium dependency) transcription factor and HDA1 of *S. cerevisiae* belong to the major classes 1 and 2 of the ‘‘classical’’ HDACs, respectively. Recently, multiple effects of the inactivation of *hda1* ortholog on the expression of secondary metabolite genes has been reported for a number of fungal species (Lee et al., 2009; Shwab et al., 2007; Tribus et al., 2005).

Here, we have demonstrated that ortholog of the class 2 histone deacetylase *hda1* of *S. cerevisiae* (Pc21 g14570) is a key regulator of the secondary metabolism in the filamentous fungus *P. chrysogenum*. By means of the transcriptional and metabolite profiling of the individual gene deletion mutants, the role of HdaA in production of the new metabolite, conidial pigmentation, as well as the broad influence of HdaA on the expression of the SM gene clusters have been shown. Furthermore, we demonstrated that transcriptional crosstalk between sorbicillinoids biosynthesis and other SM genes in this fungus is mediated by HdaA.

## 2 MATERIAL AND METHODS

### 2.1 Strains, media, and growth conditions

*Penicillium chrysogenum* DS68530 was provided by DSM Sinochem Pharmaceuticals (Delft, The Netherlands). The strains: ∆*hdaA_DS68530*, ∆*pks17*, and overexpression mutant *oepks17* were derived from DS68530. ∆*hdaA_DS68530Res13* was derived from *DS68530Res13* (Sorb407) (Guzman‐Chavez et al., 2017; Salo et al., 2016) (Table 1). Liquid YGG medium (400 ml KCl‐glucose, 100 ml 5× buffered Yeast Nitrogen Base (YNB), 10 ml fresh 10% yeast extract) was used for preculturing the conidia for 24 hr before inoculation into secondary metabolite production medium (SMP; (Ali et al., 2013)). Solid R‐agar medium (6 ml/L glycerol, 7.5 ml/L beet molasses, 5 g/L yeast extract, 18 g/L NaCl, 50 mg ml^−1^ L^−1^ MgSO_4_·7H_2_O, 60 mg/L KH_2_PO_4_, 250 mg/L CaSO_4_, 1.6 ml/L NH_4_Fe(SO_4_)_2_ (1 mg/ml), Fe(SO_4_)_2_ 12H_2_O, 10 mg/L CuSO_4_·5H_2_O, and 20 g/L agar was used for culturing the conidia and for secondary metabolites production on plate (Kovalchuk, Weber, Nijland, Bovenberg, & Driessen, 2012). All cultivations were performed at 25°C in semi‐dark conditions. Liquid culturing of the conidia was performed in 25 ml of YGG or SPM media in 100 ml flasks shaken at 200 rpm (Guzman‐Chavez et al., 2017).

**Table 1:** Strains used in this study

Strain	Genotype	Source
DS68530 (AFF407)	0 Penicillin BGC, *Δku70* Sorbicillinoid nonproducer	DSM Sinochem Pharmaceuticals
DS68530Res13 (Sorb407)	0 Penicillin BGC, *Δku70*, AmdS marker free, Sorbicillinoid producer, SorA (F146L)	Guzman‐Chavez et al., (2017)
Strains derived from DS68530		
∆*hdaA_DS68530*	AmdS marker Sorbicillinoid nonproducer	This study
*Δpks17*	AmdS marker Sorbicillinoid nonproducer	This study
*oepks17*	AmdS marker, *pcbC::Pc21 g16000* Sorbicillinoid nonproducer	This study
Strains derived from DS68530Res13		
∆*hdaA_DS68530Res13*	AmdS marker, sorbicillinoid producer*,* SorA (F146L)	This study

### 2.2 Plasmids construction

All the plasmids in this study were constructed using the modified Gateway cloning protocol (Invitrogen, California, USA) published previously (Kovalchuk et al., 2012). 5‐′ and 3‐′ fragments for the deletion cassette were amplified with Phire Hot Start II PCR Master Mix (Thermo Fisher Scientific, San Jose, CA) using specific primers and cloned into corresponded Gateway donor vectors pDONR P4‐P1R and pDONR P2R‐P3, respectively, using BP clonase II enzyme mix (Invitrogen). The resulting plasmids were purified from kanamycin‐resistant *E. coli* Dh5α transformants and subsequently recombined with the Gateway destination vector pDEST R4‐R3 and pDONR221‐AMDS for in vitro recombination using LR clonase II enzyme mix. For expression, the modified pDONR221‐AMDS plasmid was used. In this construct the *pcbC* (isopenicillin synthase) promoter region was ligated downstream of the *amdS* gene. After incubation, the reaction mixture was transformed to *E. coli* Dh5α and the final plasmids were isolated from the ampicillin‐resistant transformants (Salo et al., 2016).

### 2.3 Fungal transformation

For all the transformations, 5 μg of plasmid were digested with the suitable restriction enzymes. The linearized plasmid was used to transform protoplasts isolated from *P. chrysogenum* as described previously (Kovalchuk et al., 2012; Weber, Kovalchuk, Bovenberg, & Driessen, 2012). After 6 days of growth at 25°C on 0.1% acetamide selection plates, the correct transformants were screened by colony PCR using Phire Plant Direct Kit (Life Technologies, USA) (Guzman‐Chavez et al., 2017) and following the manufacturer's instructions. PCR product was digested with *SalI* restriction enzyme (restriction sites only present in positive transformants). Selected colonies were purified by three rounds of selection in R‐Agar. Correct transformants were validated by sequencing the amplified integration site from gDNA (Figure S1). All the primers used are described in Table S1.

### 2.4 Southern blot analysis

The 3′ downstream region of the *hdaA* gene was used as a probe and amplified by PCR with primer set listed in Table S1. The probe was labeled with digoxigenin using the HighPrime Kit (Roche Applied Sciences, Almere, The Netherlands). gDNA (10 μg) was digested with appropriate restriction enzyme and separated on 0.8% agarose gel. After equilibration in 20× SSC buffer (3 mol/L sodium chloride; 0.3 mol/L sodium citrate) the DNA was transferred overnight onto Zeta‐probe positively charged nylon membrane (BioRad, Munchen, Germany). Blots were treated with anti‐DIG‐alkaline phosphatase antibodies and supplemented with CDP‐star (Roche Applied Sciences). The fluorescence signal was measured with a Lumi Imager (Figure S1) (Fujifilm LAS‐4000, Fujifilm Co. Ltd, Tokio, Japan) (Guzman‐Chavez et al., 2017; Salo et al., 2016).

### 2.5 Genomic DNA extraction

The total genomic DNA (gDNA) was isolated after 96 hr of cultivation in YGG liquid medium using an adapted yeast genomic DNA isolation protocol (Harju, Fedosyuk, & Peterson, 2004). The mycelium was broken in a FastPrep FP120 system (Qbiogene, Cedex, France).

### 2.6 Total RNA extraction and cDNA synthesis

Total RNA was isolated from colonies of fungal mycelium that was grown on solid R‐agar medium and SMP medium for 7 and 3 days, respectively. The Trizol (Invitrogen) extraction method was used, with additional DNAse treatment using the Turbo DNA‐free kit (Ambion, Carlsbad, CA, USA). Total RNA concentration was measured with a NanoDrop ND‐1000 (ISOGEN, Utrecht, The Netherlands). For the synthesis of cDNA by iScript cDNA synthesis kit (Bio‐Rad, Munchen, Germany), 500 ng of RNA per reaction was used (Nijland et al., 2010).

### 2.7 qPCR analysis

The primers used for expression analysis of the 20 PKSs, 11 NRPSs, the sorbicillinoids gene cluster [*Pc21 g05050* (*sorR1*), *Pc21 g05060* (*sorC*), *Pc21 g05070* (pks12; *sorB*), *Pc21 g05080* (pks 13; *sorA*), *Pc21 g05090* (*sorR2*), *Pc21 g05100* (*sorT*), and *Pc21 g05110* (*sorD*) (Guzman‐Chavez et al., 2017; Salo et al., 2015)], the genes of putative DHN‐melanin cluster [*Pc21 g16380* (*abr1)*,* Pc21 g16420* (*arp1*), *Pc21 g16430* (*arp2*), *Pc21 g16440* (*ayg1*), *Pc22 g08420* (*abr2*)], and the chrysogine biosynthetic gene cluster [*Pc21 g12570* (*chyE*), *Pc21 g12590* (*chyH*), *Pc21 g12600* (*chyC*), *Pc21 g12610* (*chyM*), *Pc21 g12620* (*chyD*), *Pc21 g12630* (*nrps 9; chyA*), *Pc21 g12640* (*chyR*) (Viggiano et al., 2017)] are shown in the Table S1. Primers were designed at both sides of the introns in order to be able to discriminate between the amplification on gDNA and cDNA. For expression analysis, the γ‐actin gene (*Pc20 g11630*) was used as a control for normalization (Nijland et al., 2010). A negative reverse transcriptase (RT) control was used to determine the gDNA contamination in the isolated total RNA. The expression levels were analyzed, in duplicate, with a MiniOpticon system (Bio‐Rad) using the Bio‐Rad CFX^™^ manager software, with which the threshold cycle (ct) values were determined automatically by regression. The SensiMix SYBR Hi‐ROX kit (Bioline, Australia) was used as a master mix for qPCR. The following thermocycler conditions were applied: 95°C for 10 min, followed by 40 cycles of 95°C for 15 s, 55°C for 30 s, and 72°C for 30 s. Subsequently, a melting curve was generated to determine the specificity of the qPCRs (Nijland et al., 2010; Weber, Polli, Boer, Bovenberg, & Driessen, 2012). The expression analysis was performed for two biological samples with two technical replicates. The analysis of the relative gene expression was performed through the 2^−ΔΔCT^ method (Livak & Schmittgen, 2001).

### 2.8 Secondary metabolite analysis

The extraction of secondary metabolites from solid R‐agar medium for HPLC and MS analysis was done by the modified microscale extraction procedure for standardized screening of fungal metabolite production in cultures (Smedsgaard, 1997). A plug of the agar medium (5 mm in diameter) was taken for extraction from the middle of the colony obtained after 10 days of growth. The extraction mixture (0.5 ml) contained methanol‐dichloromethane‐ethyl acetate in a ratio of 1:2:3 (v/v). The plugs were extracted ultrasonically in 1 ml glass tubes during 60 min. The liquid extract was transferred to a fresh tube and dried under vacuum using a SpeedVac^™^ vacuum concentrator (Eppendorf, Hamburg, Germany) for 30 min. The dry pellet was redissolved in a 1:1 solution of methanol in water, filtered via 0.2 μm PTFE syringe filter and used for HPLC and MS analysis. Samples from liquid cultures in SMP medium were collected at 3 and 5 days, whereupon the supernatants were centrifuged for 5 min at 23 000 × g, previously filtered through a 0.2 μm PTFE syringe filter (Guzman‐Chavez et al., 2017; Salo et al., 2016). Secondary metabolites were analyzed with a Shimadzu HPLC system coupled with photodiode array detector (PDA) and it was performed as described previously (Salo et al., 2016). Metabolite analysis was performed with two biological samples with two technical duplicates. Reserpine was used as internal standard.

### 2.9 Scanning electron microscopy

Conidia were immobilized on glass cover slips and fixed with 2% glutaraldehyde for 1 hr followed by washing with cacodylate buffer (pH 7.4). Samples were incubated with 1% OsO_4_ in 0.1 mol/L cacodylate buffer during 1 hr and washed with MQ water. The immobilized spores were dehydrated with a concentration gradient of 30, 50, and 70% of ethanol within 30 min followed by three steps of final dehydration with 96% ethanol within 45 min. Next, the samples were incubated in 100% ethanol/tetramethylsilane (TMS) 1:1(v/v) for 10 min followed by 15‐min incubation with pure TMS and air‐dried. Dried samples were coated with 2 nm Pd/Au using Leica EM SCD050 sputter coater and analyzed with SUPRA 55 FE‐SEM (Carl Zeiss, Jena Germany) at 2 kV.

### 2.10 Oxidative stress assay

Fungal conidia of 7‐day grown mycelium were resuspended in 1 ml water containing 0.05% of Tween‐20 to prevent aggregation. The equal amount of the spores (3 × 10^3^ spores per ml) in solution were adjusted by series of dilutions and measured with Bürker‐Türk counting chamber using Olympus CX20^™^ light microscope (Olympus, Hamburg, Germany). A conidial suspension (100 μl) was used for inoculation to obtain approximately 300 germination events per control plate in the assay. R‐agar sporulation medium with increasing concentrations of hydrogen peroxide from 0.5 to 3.5 mmol/L was used in this assay. To prepare each plate, the corresponding amounts of hydrogen peroxide have been mixed with 25 ml of R‐agar medium before solidification to provide equal distribution of the supplement in the plate. The experiment was performed twice using two sets of the hydrogen peroxide supplemented R‐Agar plates as technical replicas.

### 2.11 Other methods

To study the effect of sorbicillinoids on the secondary metabolism of *P. chrysogenum*, the feeding experiment has been performed as it was reported previously (Guzman‐Chavez et al., 2017). The preculture of the strain DS368530 was grown on YGG medium for 24 hr and subsequently used (3 ml) to supplement 20 ml of fresh SMP medium. The filtered supernatant (2 ml) obtained from the growth medium of the strain DS68530Res13 grown in SPM for 3 days was collected as the source of sorbicillinoids used for the feeding experiment. The control culture was supplemented with the supernatant derived from the non‐sorbicillinoids‐producing strain DS68530.

## 3 RESULTS

### 3.1 Deletion of the *hdaA* gene

The gene, *Pc21 g14570* of *P. chrysogenum* encodes an ortholog to the *hda1* histone deacetylase gene of *Saccharomyces cerevisiae*. This gene, termed *hdaA*, was deleted from the chromosome in order to investigate its effect on development and secondary metabolite production. The complete *hdaA* gene was replaced by the acetamidase (*amdS*) selection marker gene. A standard protocol was used for cloning of the corresponded pHdaA deletion plasmid (Kovalchuk et al., 2012) containing the 3′ and 5′ flanking regions of the *hdaA* open reading frame. Protoplasts of the *amdS* marker‐free strain DS68530 and DS68530Res13 that both lack all copies of the penicillin biosynthesis cluster were used for transformation to simplify the detection of other secondary metabolites. Acetamide supplemented medium (0.1% AMDS) was used for the positive selection of transformants, and the correct inactivation of the *hdaA* gene was validated by sequencing the locus of the insertion (Figure S1).

### 3.2 Effect of the *hadA* deletion on the expression of secondary metabolite genes

To examine the effect of inactivation of *hdaA* on the transcription of secondary metabolite genes, the expression of all 20 PKS and 11 NRPS genes was examined using quantitative real‐time PCR analysis. RNA was isolated from the mycelium of the deletion and the parental strains grown on SMP medium for 3 days. The related supernatant fractions obtained after 3 and 5 days of culture growth were used for secondary metabolite profiling (see below). The qPCR analysis of the various secondary metabolite genes was performed using primers listed in Table S1. Out of the 31 analyzed secondary metabolite genes, the expression of eight genes was dramatically altered in *∆hdaA* mutants from the different genetic backgrounds (sorbicillinoids producer and none producer strains).

**Figure 1:**
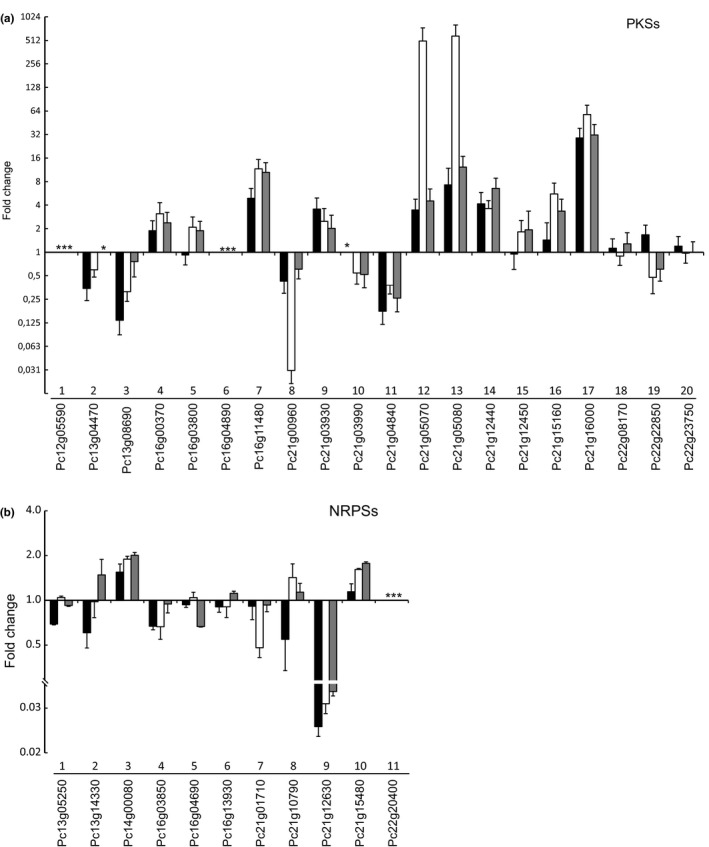
Relative expression of all secondary metabolite genes in *∆hdaA* mutants. (a) Polyketide synthases genes. (b) Nonribosomal peptide synthetase. Genes are grouped according to the genome annotation number. Samples were taken after 3 days of growth on SMP medium. Strains: DS685Res13 (black bars), ΔhdaA_DS68530Res13 (white bars), ΔhdaA_DS68530 (gray bars). (*) Indicates lack of expression. Data are shown as fold change relative to *P. chrysogenum* DS68530. (*∆hdaA*/DS68530). Error bars indicate the standard deviation of two biological with two technical replicates

An up to 500‐fold increase in expression occurred for the PKS enzymes SorB *(pks12; Pc21 g05070*) and SorA (*pks13; Pc21 g05080*) in the sorbicillinoids‐producing strain, while the transcript levels of the corresponding genes in the *∆hdaA* mutant that is not able to produce sorbicillinoids, was only 12‐fold higher. Interestingly, the deletion of *hadA* showed the same positive impact in the expression levels of *pks* 4, 7, 8, 11, and 17 (*Pc16 g00370, Pc16 g11480, Pc21 g00960, Pc21 g04840, Pc21 g16000*, respectively), compared to the parental sorbicillinoids producer strain (Figure 1a). *Pks 7* and *pks 17* expression levels were increased 11‐ and 58‐fold, respectively, while the expression of *pks 8* was reduced 33‐fold. Also, the expression of two NRPS genes *nrps 3* and *chyA* (*Pc13 g08690* and *Pc21 g12630*, respectively) were significantly altered (Figure 1b). The *chyA* (*nrps9*) gene that encodes for a dipeptide synthase that belongs to the chrysogine biosynthetic gene cluster (BGC) (Viggiano et al., 2017) was 25‐fold downregulated. In the genome of *P. chrysogenum,* secondary metabolite genes are distributed over the four chromosomes. However, in particular, genes that localize to chromosome 2 were influenced by the *hdaA* deletion, except for *pks7* and *nrps3* that localize at the opposite ends of chromosome 1. For the remainder of the secondary metabolite genes no transcriptional response was observed (Figure 1a).

### 3.3 Epigenetic activation of the sorbicillinoids biosynthesis gene cluster

**Figure 2:**
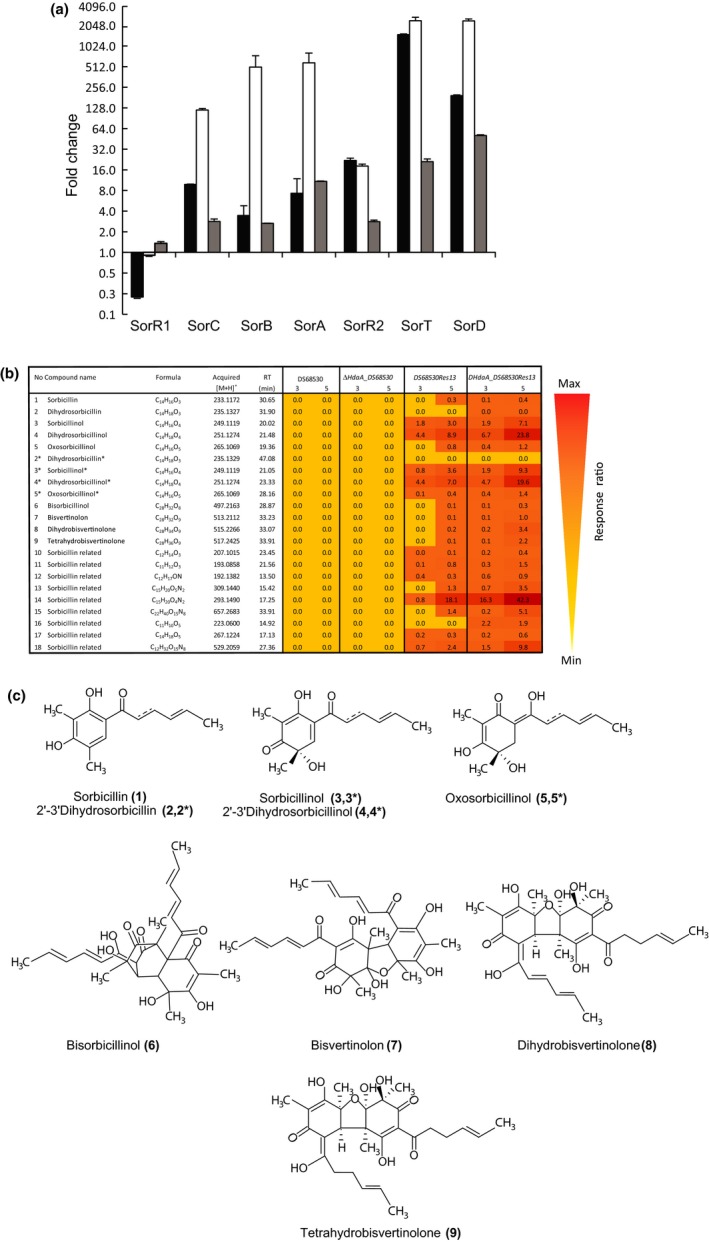
Transcription and metabolite profile analysis of the activated sorbicillinoids biosynthetic gene cluster in the *∆hdaA* mutant. (a) Quantitative real‐time PCR analysis of the sorbicillinoids BGC. Strains: DS685Res13 (black bars), ΔhdaA_DS68530Res13 (white bars), ΔhdaA_DS68530 (gray bars). Samples were taken after 3 days of growth on SMP medium. Data are shown as fold change relative to *P. chrysogenum* DS68530 (*∆hdaA*/DS68530). (b) Response ratio of the sorbicillinoids concentrations in the supernatant of the indicated *P. chrysogenum* strains. Samples were collected after 3 and 5 days of growth in SPM medium. (c) Sorbicillinoids‐related compounds (with known chemical structure) detected in this study. Reserpine was used as internal standard for normalization. The mass‐to‐charge ration (*m/z*) of the protonated metabolites, retention time, and empirical formulas are described. (*) Indicates an isomer of the known sorbicillinoids. Error bars indicate the standard deviation of two biological replicates with two technical replicates

The genes belonging to the sorbicillinoids BGC (Guzman‐Chavez et al., 2017) were highly upregulated in the *ΔhdaA* strain (Figure 2a; **∆** *hdaA_DS68530Res13* strain). The *sorC* gene showed an increase in the expression levels of more than 100 times, while *sorD* and *sorT* were 2500‐fold upregulated relative to the DS68530 strain. Overexpression of this BGC resulted in the high‐level production of sorbicillinoids in the supernatant fraction (Figure 2b). The production of sorbicillinol [3,3*] and dihydrosorbicillinol [4,4*] ‐ the main products of the pathway, increased up to 2.5‐folds in *ΔhdaA* strain. However, the most significant changes were related to the downstream intermediates of the pathway. For instance, at day 5, the levels of tetra‐ and dihydrobisvertinolone [8;9] were 17‐ and 22‐fold higher with the *hdaA* gene deletion strain compared to the parental strain, while production of sorbicillinoids was detected one day earlier in fermentation (data not showed). Importantly, the *hdaA* deletion (∆*hdaA_DS68530*) also enhanced the expression of the sorbicillinoids BGC in the strain that contains a defect copy of the *sorA* gene (Figure 2a) although the effect was not as strong as in the sorbicillinoids‐producing strain(**∆** *hdaA_DS68530Res13* strain). These observations are consistent with previous findings that sorbicillinoids act as autoinducers (Guzman‐Chavez et al., 2017) and further demonstrate that HdaA silences the expression of this BCG.

**Figure 3:**
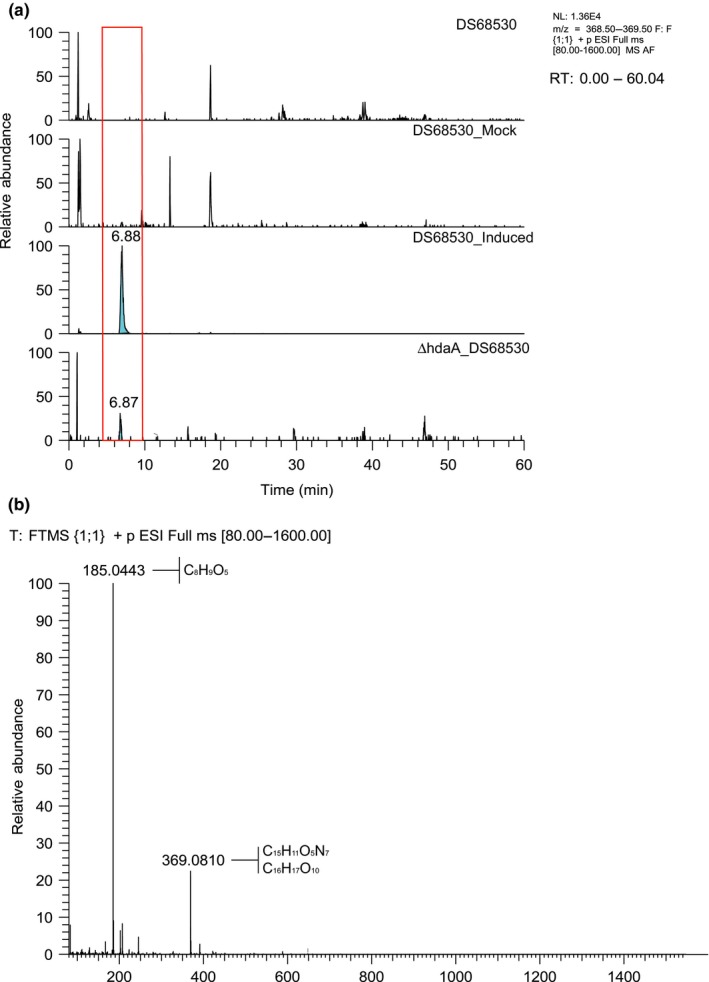
(a) LC‐MS extracted ion chromatogram of the extracellular metabolite spectrum of different strains after 5 days of growth in SPM medium. (b) LC‐MS spectra containing the empirical chemical formulas and calculated exact mass (<2.0 ppm). Data obtain from extracted ion chromatogram in positive mode

Considering the significant transcriptional deregulation of the secondary metabolite genes in the *ΔhdaA* strain and in particular the expression of functionally uncharacterized PKSs and NRPSs genes, metabolic profiling was employed to search for novel compounds. Indeed, an unknown compound was detected at elevated levels in the *ΔhdaA* strain that does not produce sorbicillinoids (∆*hdaA_DS68530*). This compound has a m/z [M+H]^+^ of 369.0810 and a retention time (RT) of 6.88 min. Interestingly, the same compound was also present in the culture broth of the DS68530 strain that was supplemented with sorbicillinoids derived from a 3‐day culture of DS68530Res13 strain (Figure 3). The identity of this compound is unknown.

### 3.4 HdaA regulates the transcription of the chrysogine biosynthetic gene cluster

**Figure 4:**
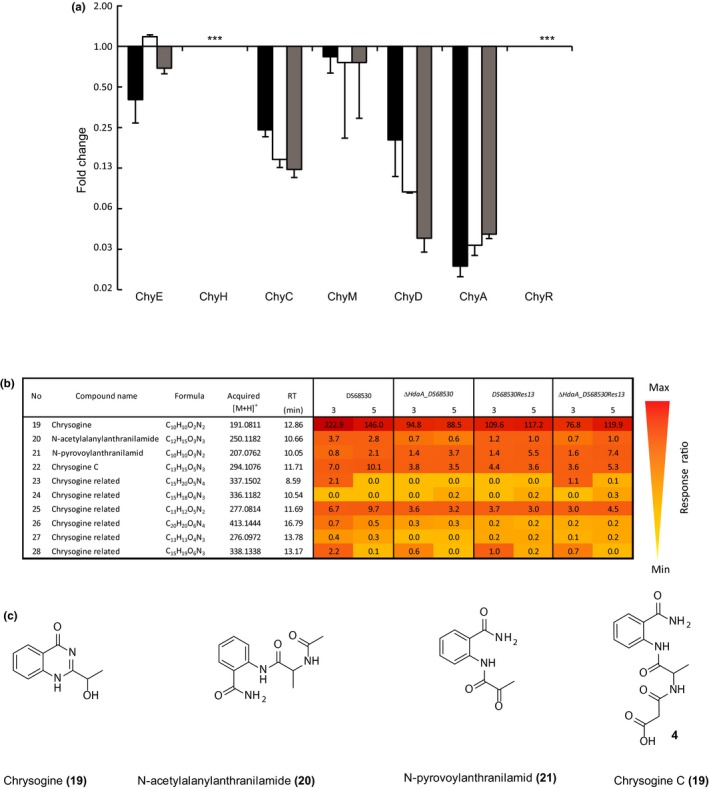
Transcriptional and metabolite profile analysis of chrysogine biosynthetic gene cluster in *∆hdaA* mutant. (a) Quantitative real‐time PCR analysis of chrysogine gene cluster. Strains: DS685Res13 (black bars), ΔhdaA_DS68530Res13 (white bars), ΔhdaA_DS68530 (gray bars). Samples were taken after 3 days of growth on SMP medium. Data are shown as a fold change relative to *P. chrysogenum* DS68530 (*∆hdaA*/DS68530). (*) Indicates nondetected expression under the tested strain. (b) Response ratio on the concentration of the chrysogine‐related compounds in the culture broth of the indicated *P. chrysogenum* strains. Samples were collected after 3 and 5 days of growth in SPM medium. (c) Chrysogine‐related compounds (with known chemical structure) detected in this study. Reserpine was used as internal standard for normalization. The mass‐to‐charge ration (*m/z*) of the protonated metabolites, retention time, and empirical formulas are described. Error bars indicate the standard deviation of two biological replicates with two technical replicates

Pc21 g12630 encodes an NRPS (*chyA*) that is involved in chrysogine production (Viggiano et al., 2017) and its expression is downregulated in the *hdaA* deletion strain. *ChyA* is part of a cluster of seven genes that in addition specifies a malonyl transferase (*chyE*;* Pc21 g12570*), two asparagine synthetase (*chyC*,* chyD* [*Pc21 g12600 Pc21 g12620*]), two hypothetical proteins involved in oxidation reactions (*chyH, chyM* [*Pc21 g12590*,* Pc21 g12610*]), and a putative regulator (*chyR*,* Pc21 g12640*)). The expression of the cluster was analyzed by qPCR revealing the downregulation of the entire BGC in the *hdaA* mutants. However, downregulation was also observed with the sorbicillinoids production DS68530Res13 strain independent of the *hdaA* deletion. The transcriptional levels of *chyA* and *chyD* were reduced up to 25‐fold, while gene expression of *chyE*,* chyC*, and *chyM* were lowered 2.8‐ and 2‐fold, respectively (Figure 4a). To investigate the effect of the *hdaA* gene deletion on the production of chrysogine, the reference strain DS68530, DS68530Res13 and *∆hdaA* strains were grown for 3 and 5 days in SMP medium. Samples of the culture broth were filtered and analyzed by LC‐MS. At day 3, chrysogine production was reduced twofold for the ∆*hdaA* mutants and DS6830Res13 compared to the reference DS68530 strain in line with the qPCR data (Figure 4a,b). Likewise, at day 5, also most of the chrysogine‐related compounds were produced at lower levels. Taken together, these results indicate that the chrysogine BGC is not only subjected to epigenetic activation, but also suppressed by the production of sorbicillinoids.

### 3.5 HdaA regulates the DHN‐melanin BGC involved in pigment formation

**Figure 5:**
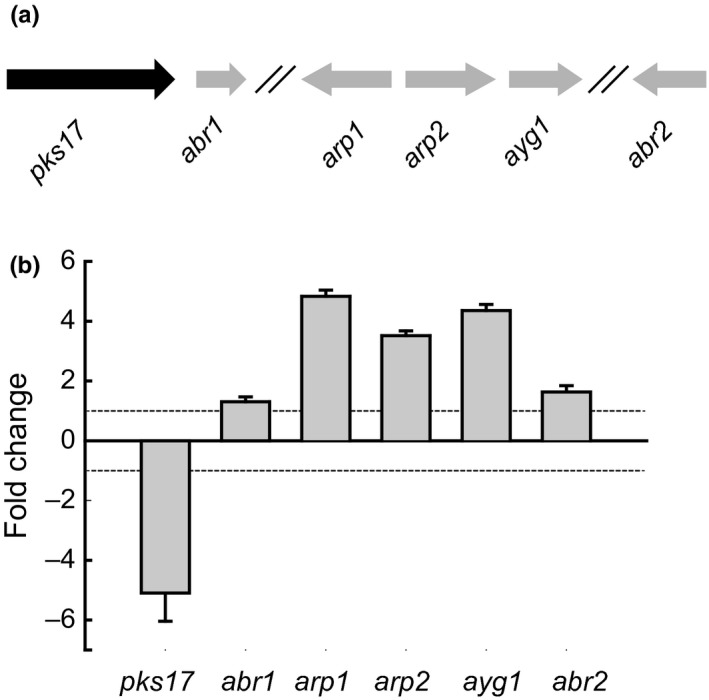
(a) Schematic representation of the DHN‐melanin biosynthetic gene cluster. (b) Quantitative real‐time PCR analysis of the DHN‐melanin BGC in ∆hdaA_DS68530 mutant. Samples were taken after 7 days of growth on solid R‐agar medium. The expression data are shown as fold change (*∆hdaA*/DS68530). Error bars indicate the standard deviation of two biological replicates with two technical replicates

**Figure 6:**
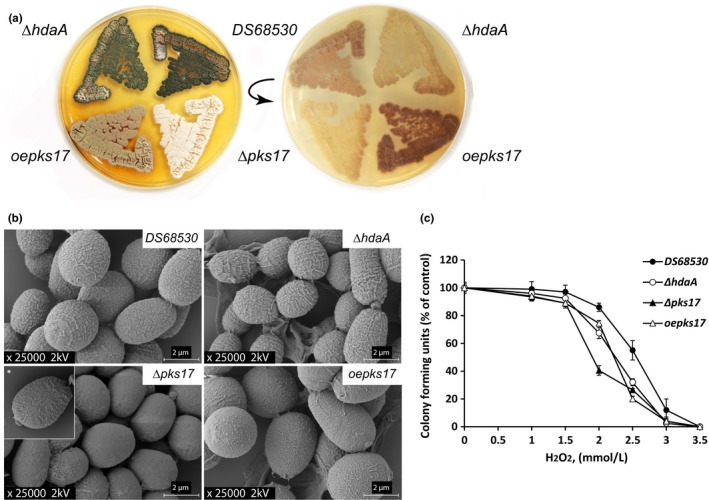
(a) Pigmentation differences between *DS68530*,* ∆hdaA_DS68530, ∆pks17*, and *oepks17* strains. Top (left) and bottom (right) of the plate. The picture has been taken after 14 days of growth grown on solid R‐agar medium. (b) Scanning electron microscopy of the conidia of strain DS68530*, ∆hdaA, ∆pks17* (albino mutant), and the *oepks17* mutant. The cell wall surface of the conidia for *∆pks17* and *oepks17* strains is shown. The *∆hdaA* mutant displays a more pronounced relief of the conidial surface ornamentation in comparison to the reference strain DS68530. (c) Percent survival of conidia grown in the presence of hydrogen peroxide on R‐agar medium. Germinated colonies were counted after 5 days of growth. Error bars indicate the standard deviation of two biological replicates with two technical replicates

Pigmentation in filamentous fungi is often attributed to the dihydroxynaphthalene (DHN)‐melanin BGC that typically consists of six genes including a PKS (Tsai, Chang, Washburn, Wheeler, & Kwon‐Chung, 1998; Tsai, Washburn, Chang, & Kwon‐Chung, 1997). The DHN‐melanin biosynthetic pathway was described initially for *Verticillium dahliae* and *Wangiella dermatitidis* (Bell, Puhalla, Tolmsoff, & Stipanovic, 1976; Geis, Wheeler, & Szaniszlo, 1984). The pentaketide origin of fungal melanins is common in other melanized fungi (Langfelder, Streibel, Jahn, Haase, & Brakhage, 2003; Wheeler et al., 2008). In *A. fumigatus*, the polyketide product of the PKS Alb1p, the heptaketide naphthapyrone YWA1, requires the enzymatic post‐PKS conversion into the pentaketide 1,3,6,8‐tetrahydroxynaphthalene (T4HN) via hydrolytic polyketide shortening by Ayg1p (Fujii et al., 2004). This enzymatic step is absent in *C. lagenarium* where the pentaketide T4HN is a direct product of PKS1 (Fujii et al., 1999). Next, it is reduced to scytalone via the T4HN reductase Arp2p, followed by dehydration to 1,3,8‐trihydroxynaphthalene (T3HN) by the scytalondehydratase Arp1p. The following reduction to vermelon is Arp2 dependent but the presence of other specific reductase(s) caring this reaction has been also proposed for *Aspergilli* and other fungi (Tsai, Wheeler, Chang, & Kwon‐Chung, 1999; Wang & Breuil, 2002). The dehydration of vermelon to 1,8‐dihydroxynaphthalene (DHN) is Arp1p dependent. The resulting DHN molecules are further polymerized to the structurally diverse melanins. This final enzymatic step involves the multicopper oxidase Abr1p and laccase Abr2p (Hamilton & Gomez, 2002; Jacobson, 2000; Langfelder et al., 2003). Screening of the genome sequence of *P. chrysogenum* indicates the presence of the corresponding ortholog genes of the DHN‐melanin BGC: *abr1* (*Pc21 g16380*), *arp1* (*Pc21 g16420*), *arp2* (*Pc21 g16430*), *ayg1* (*P21 g16440*), *abr2* (*P22 g08420*) and associated *pks17* (*pcAlb1*,* Pc21 g16000*) were found partially clustered in the genome. To examine the role of HdaA in the biosynthesis of DHN‐melanin in conidial pigmentation, the ∆*hdaA* mutant and DS68530 strains (no sorbicillinoids producers) were grown on solid R‐agar medium for 10 days, which resulted in a major decrease in the green conidial pigmentation in the ∆*hdaA* mutant as compared to the reference strain. qPCR analysis of the putative DHN‐melanin BGC indicated the fourfold downregulation of *pks17* in the ∆*hdaA* mutant, while *arp1*,* arp2*, and *ayg1* were fourfold upregulated. Expression of *abr1* and *abr2* was not significantly changed (Figure 5a,b). To determine if pks17 is involved in conidial pigment biosynthesis, the *pks17* gene was deleted and overexpressed in order to identify the related polyketide product. A gene inactivation strain was obtained as described earlier (see materials and methods section) using primers listed in Table S1. The resulting ∆*pks17* mutant displayed an albino phenotype of the conidia while grown on sporulating R‐agar medium (Figure 6a). For the overexpression, *pks17* was placed under control of the isopenicillin N synthetase (*pcbC)* gene promoter and integrated into the genome. As a result, a 10‐fold increase in the transcript level was obtained as compared to the reference strain. The solid medium grown mutant featured a deficient coloring of the conidia and intense pigmentation of the bottom surface of the colony (Figure 6a). To identify the accumulated product, extracted R‐agar medium of a 7‐day grown culture was analyzed by the full mass range LC‐ESI‐MS Orbitrap (Thermo Fisher Scientific). The overproduced metabolite with the exact mass *m/z* [M‐H]^−^ 275.06 has been detected. The elemental composition of the deprotonated molecule has been calculated as C_14_H_11_O_6_ using build‐in Qual Browser tool of Excalibur 2.1 (Thermo Fisher Scientific) with 0.35 ppm accuracy. The observed mass, calculated elemental formula, and characteristic fragmentation pattern belongs to the known heptaketide YWA1 of *A. nidulans* produced by the highly homologous PKS wA that is involved in the conidial DHN‐melanin biosynthetic pathway (Figure 3a). In addition to the analyzed strains, the sorbicillinoids producer strains (mutant and parental) were also grown on SMP medium for 3 and 5 days. Transcriptional analysis showed an overexpression of the genes that belong to putative DHN‐melanin BGC in all the tested strains. An up to 50‐fold increase was observed in the Pks17 overproducing strain (Figure 4a). YWA1 could only be detected in the culture supernatant of the Pks17‐overproducing strain, and was not found in parental strain.

### 3.6 Role of pks17 in conidia formation and tolerance to oxidative stress

Melanins are important components of the conidial cell wall and its integrity. They play an essential role in physical properties of the spores like surface interaction, hydrophobicity, and virulence in pathogenic fungal species. The effect of *hdaA* deletion on the conidial surface in *P. chrysogenum* was examined using scanning electron microscopy. The conidia of the reference DS68530, ∆*hdaA_DS68530*, ∆*pks17*, and overexpression mutant *oepks17* were isolated from colonies grown for 7 days on the sporulating R‐agar medium. The reference *DS68530* exhibited a typical tuberous surface of the conidia, while the spores of albino mutant ∆*pks17* were smooth. The texture of the *∆hdaA_DS68530* spores surface was more pronounced compared to the parental strain but without dramatic changes of the conidial cell wall appearance (Figure 6b). These data suggest that Pks17 is involved in pigment formation and influences the morphology of the conidia in *P. chrysogenum*. The Pks17 protein was renamed Alb1 according to the nomenclature of *A. fumigatus*.

Aside from the mechanical properties of the pigments (Howard, Ferrari, Roach, & Money, 1991) and their role as pH buffering systems, the scavenging of reactive oxygen species is an important feature supporting UV and thermo‐tolerance and pathogenicity of the conidia (Jacobson, Hove, & Emery, 1995; Kawamura et al., 1997; Romero‐Martinez, Wheeler, Guerrero‐Plata, Rico, & Torres‐Guerrero, 2000).The *P.chrysogenum ∆hdaA_DS68530* mutant was grown on hydrogen peroxide supplemented medium for 5 days to verify the ability of the conidia to survive oxidative stress conditions in the absence of pigmentation. The survival rate was decreased by 20% when the *∆hdaA* strain was exposed to 2 mmol/L of hydrogen peroxide in the media. Under the same conditions, the survival rate of the *∆pks17* (*∆alb1)* was reduced more than 50%. There was no enhanced survival observed for the oepks17 overexpression mutant (Figure 6c).

## 4 DISCUSSION

Recent genome sequencing and metabolite analysis studies revealed that the majority of the potential biosynthetic gene clusters (BGCs) present in genomes of filamentous fungi are silent or expressed at a low level under standard laboratory conditions. These nonexpressed BGCs represent a potential untapped source of novel bioactive molecules. Activation of the secondary metabolites production via deletion or chemical inhibition of histone deacetylases was recently reported for filamentous fungi as an effective tool for silent SM gene clusters’ activation and identification of new metabolites with potential pharmaceutical properties (Fisch et al., 2009; Lee et al., 2009; Shwab et al., 2007; Tribus et al., 2005). Here, we have examined the effect of chromatin modification on the expression of the secondary metabolism‐associated genes and products in the fungus *P. chrysogenum*. In this work, the *P. chrysogenum hdaA* gene encoding an ortholog of the class 2 histone deacetylase *Hda1* of *S. cerevisiae* was deleted. The deletion mutant showed significant changes of secondary metabolite gene expression including PKS and NRPS genes with known and unknown function (Figure 1). In *ΔhdaA* mutants, the transcriptional levels of the sorbicillinoids BGC were significantly increased. *SorA* and *sorB* genes, which encodes for the two polyketide synthases (highly reducing and nonreducing, respectively) involved in the sorbicillinoids pathway (Salo et al., 2016) were overexpressed in the *ΔhdaA*_DS38530 strain. Interestingly, overexpression of the sorbicillinoids BGC was also observed in the DS38530Res13 strain, in which *hdaA* was not deleted. This has been attributed to a complex regulation mechanism that involves sorbicillinoids as auto inducers (Guzman‐Chavez et al., 2017). This phenomenon could potentially also involve HdaA altering the chromatin landscape (Brosch et al., 2008), since the deletion of the *hdaA* gene in the sorbicillinoids producer strain (Figure 2a) resulted in an additive effect (up to 500‐fold) on the expression levels of both *pks* genes. Indeed all genes that belong to the BGC showed a similar trend likely because the chromatin state can module gene expression by improving the binding of transcriptional factors (Macheleidt et al., 2016). These observations agree with the pronounced effect of the *hdaA* deletion on sorbicillinoids production (Figure 2b), and the earlier onset of production (data not shown). In contrast, the transcript levels of the *chyA* (*nrps9*) gene of chrysogine biosynthesis were significantly (25‐fold) reduced in the *ΔhdaA* mutants but this reduction in expression was also observed in the sorbicillinoids‐producing strain (Figure 4). qPCR analysis indicated that the chrysogine BGC (Viggiano et al., 2017) was downregulated in the aforementioned strains with a corresponding decrease in chrysogine‐related metabolites in the culture supernatant [19]. Likely, the chrysogine BGS is subjected to eplgenetic activation by HdaA, but at the same time sorbicillinoids production reduces the expression of this gene cluster.

In *A. nidulans and A. fumigatus,* the homologous *hdaA* is a main contributor of histone deacetylase activity in these fungi. In *A. nidulans*, deletion of the *hdaA* gene stimulated penicillin and sterigmatocystin production but not a telomere‐distal gene cluster involved in terraquinone A biosynthesis. It was suggested, that HdaA silences the expression of subtelomeric chromosomal regions (Tribus et al., 2005). In contrast, the HdaA homolog in *A. fumigatus* was reported to activate gliotoxin biosynthesis and to repress several NRPSs including one gene of the siderophore BGC. A subtelomeric specificity of HdaA was not apparent for this fungus (Lee et al., 2009). In silico comparative analysis of the *P. chrysogenum* genome sequence revealed four chromosomes on which all BGC are distributed (Specht, Dahlmann, Zadra, Kürnsteiner, & Kück, 2014). We performed expression analysis of all 11 NRPS and 20 PKS genes in the Δ*hdaA* strain. The results show that the expression of eight secondary metabolite genes was significantly altered in the ∆*hdaA* strain including the activation of a silent PKS cluster with unknown function. It is important to stress that the particular effect seems to be restricted to chromosome 2 and the chromosome 1 extremes. The chromosome 2 region contains a remarkably large number of BGCs, comprising 15 of the 32 PKS and NRPS‐encoding genes. The few remaining BCGs are distributed throughout the other chromosomes (Figure S2). The action of HdaA thus seems mostly to be restricted to the transcriptional coregulation of a particular genomic area rich in BGCs (Van Den Berg et al., 2008).

The production of sorbicillinoids and the deletion of the *hdaA* gene, causing increased sorbicillinoids production, had similar effects on the expression of other PKS and NRPS genes (Figures 1, 2, 4) including the BGC that specifies chrysogine. One possible explanation is that sorbicillinoids might act as HdaA inhibitors, since *hdaA* was transcribed at the same levels in the sorbicillinoids producer and nonproducer strains (data not shown), while feed sorbicillinoids did not alter the transcription of hdaA in the DS68530 strain. Moreover, a novel compound was detected only when *hdaA* was deleted or when DS68530 was fed with sorbicillinoids (Figure 3). A similar phenomena occurs when *Cladosporium cladosporioides* and *A. niger* are exposed to suberoylanilide hydroxamic acid (SAHA), a HDAC inhibitor, which induces the synthesis of two new compounds, cladochrome and nygerone A, respectively (Rutledge & Challis, 2015). Our work represents the first example of a regulatory crosstalk between BGCs in *P. chrysogenum*. In *A. nidulans*, overexpression of a regulator (*scpR*) was found to activate the expression of two cryptic NRPS genes, belonging to the same cluster, as well as the induction of genes responsible for the production of the polyketide asperfuranone (Bergmann et al., 2010; Brakhage, 2012).

The deletion of the *hdaA* gene also has a functional effect in *P. chrysogenum*, since it decreases green conidial pigmentation and an altered surface structure of the spores. The function of the fourfold downregulated pks17 (Pc21 g16000) gene was elucidated via gene deletion and overexpression. This PKS enzyme shows a high similarity to the *A. nidulans* wA and *A. fumigatus* PksP proteins involved in conidial pigment biosynthesis (Van Den Berg et al., 2008). To identify the polyketide product, *pks17* was overexpressed causing the accumulation of the yellow naphtho‐γ‐pyrone (a polyketide precursor of the conidial pigment) into the medium. This indicates that *P. chrysogenum* uses the DHN‐melanin biosynthetic pathway like previously reported for *Aspergillus* (Hamilton & Gomez, 2002; Jacobson, 2000; Langfelder et al., 2003). The corresponding ∆*pks17* strain showed an albino phenotype confirming the primary role of this gene in the conidial pigmentation. In the closely related fungus *A. fumigatus* at least six genes are required for DHN‐melanin biosynthesis, which were found to be partially clustered in the genome of *P. chrysogenum*. qPCR analysis (Figure 5) showed the fourfold upregulation of the *arp1* (scytalondehydratase)*, arp2* (T4HN reductase), and *ayg1* (enzyme of hydrolytic polyketide chain shortening activity) genes, while the transcript level of *abr1* (multicopper oxidase) and *abr2* (laccase) which products catalyze the last steps of the polymerization of 1,8‐dihydroxynaphthalene were not significantly changed. Scavenging of reactive oxygen species by fungal melanins provides an important defense mechanism during growth under oxidative stress condition. We examined the effect of *hdaA* deletion on the ability of the conidia to survive high concentrations of hydrogen peroxide. An increased sensitivity of the HdaA was noted toward hydrogen peroxide, while this effect was even more pronounced for the ∆*pks17* albino mutant (Figure 6c). These results suggest that the oxidative stress response in *P. chrysogenum* involves HdaA and is mediated by the transcriptional regulation of DHN‐melanin gene cluster.

In conclusion, our results demonstrate that HdaA has a broad impact on secondary metabolism of *P. chrysogenum* at the transcriptional level causing marked changes in metabolite production. Furthermore, HdaA influences conidial pigmentation and the surface structure of spores. This work provides evidence of crosstalk between gene clusters, which impacts secondary metabolism. The presented data suggest that an epigenome approach can be successfully applied for the discovery of novel biosynthetic pathways in *P. chrysogenum*.

## CONFLICT OF INTEREST

The authors declare no conflict of interest.
