# Supplementary material for: Deregulation of secondary metabolism in a histone deacetylase mutant of Penicillium chrysogenum
Source: Microbiologyopen. 2018 Mar 24;7(5):e00598. doi: 10.1002/mbo3.598 (PMC6182556; doi:10.1002/mbo3.598)
Supplement: Supplementary file 1 [file MBO3-7-e00598-s001.docx]

**SUPPORTING INFORMATION**

**Deregulation of secondary metabolism in a histone deacetylase mutant of *Penicillium chrysogenum.***

**Fernando Guzman-Chavez, Oleksandr Salo, Marta Samol, Marco Ries, Jeroen Kuipers, Roel A.L. Bovenberg, Rob J. Vreeken, Arnold J.M. Driessen***

*Correspondence: a.j.m.driessen@rug.nl

**Table S1.** Primer list used in this study.

|  |  | **Oligonucleotides used in this study** |  |  |
| --- | --- | --- | --- | --- |
| **Primer name** | **Gene** | **Sequence 5'-3'** | **Function** | **Reference** |
|  |  |  |  |  |
|  |  |  | **qPCR PKSs/NRPSs** |  |
| PKS1F | Pc12g05590 | GCTACAGCCCTGACGCCATGG |  |  |
| PKS1R | Pc12g05590 | CTGCGCAGGTCTACATCGGTACC |  |  |
| PKS2F | Pc13g04470 | CCGAAGATGCCGGCGACGG |  |  |
| PKS2R | Pc13g04470 | CGCTGGTCTGCGATGTGGCC |  |  |
| PKS3F | Pc13g08690 | CGAGAGACCAGGATAAGGTTCTTGGC |  |  |
| PKS3R | Pc13g08690 | GGTGGTCTGTCACCACTCTTCCC |  |  |
| PKS4F | Pc16g00370 | CATGGTCAGCACCCTCAGTGCC |  |  |
| PKS4R | Pc16g00370 | CCAGGTCAGGCGTCGTACGC |  |  |
| PKS5F | Pc16g03800 | CGGGTGCTGCATAGATGTACTACGC |  |  |
| PKS5R | Pc16g03800 | GCTGGCCACGGAAGACAACGC |  |  |
| PKS6F | Pc16g04890 | CCTATTCGCGCCCTGATTATGGGC |  |  |
| PKS6R | Pc16g04890 | CGAGATTTGTCTTCACAGAACCCACC |  |  |
| PKS7F | Pc16g11480 | CACGATTTTAGCAAGTCAACCAGCGCG |  |  |
| PKS7R | Pc16g11480 | CTCGCTCTCCCAGAATGTCAAGGC |  |  |
| PKS8F | Pc21g00960 | GCCACACTCATCGGCACCACG |  |  |
| PKS8R | Pc21g00960 | GCTCCACAGAGCAACCAACCCG |  |  |
| PKS9F | Pc21g03930 | GACGTGGCCGGTGATGCCG |  |  |
| PKS9R | Pc21g03930 | GCGATGTTGCGGACGAGGCC |  |  |
| PKS10F | Pc21g03990 | CAGCGCCGAGTCCTACAGCC |  |  |
| PKS10R | Pc21g03990 | GTGGACCTTGGAGGATGTCTTGC |  |  |
| PKS11F | Pc21g04840 | CCTTGACGAATATCCGCACTCCG |  |  |
| PKS11R | Pc21g04840 | CAAGCCACAGCTGATGAAGCGC |  |  |
| PKS12F | Pc21g05070 | GTCGGAGGCAATTCGGGAAGGC |  | **Salo *et al*.,** |
| PKS12R | Pc21g05070 | GCAAAGTTCCACCACAATGCCGCG |  | **2015** |
| PKS13F | Pc21g05080 | CCGAGGATCTCCGCCAGGC |  |  |
| PKS13R | Pc21g05080 | GGTTGTGCAGGTTCCAGGTGCC |  |  |
| PKS14F | Pc21g12440 | GCACCACCATCAGCCAAAGCATACC |  |  |
| PKS14R | Pc21g12440 | CCGAGGTCCATTGGAACTATGCGC |  |  |
| PKS15F | Pc21g12450 | CCAGTTGTCTGCAGCCGGCC |  |  |
| PKS15R | Pc21g12450 | GCCCAGATCACCGCCGTACG |  |  |
| PKS16F | Pc21g15160 | CAGCCGCGTAGTTTGCCTGGC |  |  |
| PKS16R | Pc21g15160 | GCACAGTGTGCTGAGGTTACGGC |  |  |
| PKS17F | Pc21g16000 | CTTGTCATCAGCAGCCCAGAGG |  |  |
| PKS17R | Pc21g16000 | CAATTTGCGGTGGCTGAGACGC |  |  |
| PKS18F | Pc22g08170 | GGTTGATACTCCTGGGACTGAATACAG |  |  |
| PKS18R | Pc22g08170 | GCTGCTGTGGATCCATCTGCTCG |  |  |
| PKS19F | Pc22g22850 | CGGTCAACCAGGGATCCAACTGC |  |  |
| PKS19R | Pc22g22850 | CTGAAGCGGTCTCTGTGTGGCC |  |  |
| PKS20F | Pc22g23750 | CGGTAATGTCCAGCTGGCACTCG |  |  |
| PKS20R | Pc22g23750 | CTTCAGGCACTTCTGTACCGGG |  |  |
| NRPS1F | Pc13g05250 | GCAGACCTGTATCCATCGCAA |  |  |
| NRPS1R | Pc13g05250 | GGAGGCAAGTGAAGGTGTGTT |  |  |
| NRPS2F | Pc13g14330 | GCGACAGCCGCCGGAGTAACTATGG |  |  |
| NRPS2R | Pc13g14330 | GAGAGACGGGGACACGCGTGATG |  |  |
| NRPS3F | Pc14g00080 | ACGTACGCTCGAGCTGGACT |  |  |
| NRPS3R | Pc14g00080 | GCCGTCGCGTTGATAATTGG |  |  |
| NRPS4F | Pc16g03850 | TGGTTGAAAGAGGGCAGTCTC |  |  |
| NRPS4R | Pc16g03850 | CGCGAACATACACAACACCAC |  |  |
| NRPS5F | Pc16g04690 | CTTTCCAGAACAGTTGGCTGGT |  |  |
| NRPS5R | Pc16g04690 | GCTGCATCTTACCCAGGTAATTG |  |  |
| NRPS6F | Pc16g13930 | CCACCCTTGTTCAGCCGCTGAATTCC |  |  |
| NRPS6R | Pc16g13930 | GGACGAGGCGAACAACATCGGAC |  |  |
| NRPS7F | Pc21g01710 | GCTATCTCGGTGGAGGATCTTCTGTCC |  |  |
| NRPS7R | Pc21g01710 | GTGCTGCTGAGAACACGGGATTGT |  |  |
| NRPS8F | Pc21g10790 | GTGAGGCAGCTTTGTTCAACACCATT |  |  |
| NRPS8R | Pc21g10790 | TTCTGCAGCAGGCTGTCGGCCTGAG |  |  |
| NRPS9F | Pc21g12630 | GAGCCAACTCTGTTGTCTACG |  |  |
| NRPS9R | Pc21g12630 | CAGGGCAATTTGCCTCATTCTG |  |  |
| NRPS10F | Pc21g15480 | CTTGGTGGATGCAGCGAAGG |  |  |
| NRPS10R | Pc21g15480 | CTGTGAGAGAGGCTCTTGAGTA |  |  |
| NRPS11F | Pc22g20400 | TTCGCGAACATCCGAAGAAGC |  |  |
| NRPS11R | Pc22g20400 | TCGGGCGAAGACACTGTTCA |  |  |
|  |  |  |  |  |
| 12.50F | Pc21g05050 | GAAGCGTGGATAGAGACCGAAGAGAAC | **qPCR of the expressed**  **Sorbicillinoids gene cluster** |  |
| 12.50R | Pc21g05050 | GGAGCCAGACTCCGGAAAGGATACTG |  |  |
| 12.60F | Pc21g05060 | GATAGTGAGTACAAATGCGCCTGGACC |  |  |
| 12.60R | Pc21g05060 | CGTTCAATACCGGAAATGGCTAGATTCG |  |  |
| 12.70F/PKS12F | Pc21g05070 | GTCGGAGGCAATTCGGGAAGGC |  | **Salo *et al*.,** |
| 12.70R/PKS12R | Pc21g05070 | GCAAAGTTCCACCACAATGCCGCG |  | **2015** |
| 12.80F/PKS13F | Pc21g05080 | CCGAGGATCTCCGCCAGGC |  | **Guzman-** |
| 12.80R/PKS13R | Pc21g05080 | GGTTGTGCAGGTTCCAGGTGCC |  | **Chavez** |
| 12.90F | Pc21g05090 | CGTTAACTAATGACGCCACCTGTTGC |  | ***et al*., 2017** |
| 12.90R | Pc21g05090 | GGAAAATAGTATCCCCAGCGATTGGC |  |  |
| 12.100F | Pc21g05100 | CATCAGCACCGAGGTCTTCATTGTCG |  |  |
| 12.100R | Pc21g05100 | GCAACGCAATAGATGGTCAATGCCAG |  |  |
| 12.110F | Pc21g05110 | CTGCAGCACTTCAGCATGGATGAAACC |  |  |
| 12.110R | Pc21g05110 | TCGTTGTGAGACTTGGATGCTCGGACG |  |  |
| 12.120F | Pc21g05120 | CCTGCTTCTTAATCTTGCCCTGGC |  |  |
| 12.120R | Pc21g05120 | CCAAGCCGATGCCAAGAAGGAAGAG |  |  |
|  |  |  |  |  |
| s570F | Pc21g12570 | GGCAAGGGAAATGAATCCAGGTGGC | **qPCR of the Chrysogine**  **gene cluster** |  |
| s570R | Pc21g12570 | GATAGATGCCGCTTGTTCGGACC |  |  |
| s590F | Pc21g12590 | GGTTGTGGAGCTCTACGAGGCTG |  |  |
| s590R | Pc21g12590 | CTGGCAGGGCTCGTCGGTC |  |  |
| S600F | Pc21g12600 | GTAGACGCCGGTGAGACTTTGATCG |  |  |
| S600R | Pc21g12600 | CAACCTAAGCGTCTAATTTTCATCGC |  |  |
| s610F | Pc21g12610 | CCTGCATGCAGCTCCATACGAGC |  | **Viggiano** |
| s610R | Pc21g12610 | CCAACAATAGGTGGAAACAGCTCAGAC |  | ***et al*., 2017** |
| s620F | Pc21g12620 | GGAATTCGCTGGCTAACTGGTCTCG |  |  |
| s620R | Pc21g12620 | GGCATGTGGTAGACGAATTGGAGC |  |  |
| s630F/ NRPS9F | Pc21g12630 | GAGCCAACTCTGTTGTCTACG |  |  |
| s630R/ NRPS9R | Pc21g12630 | CAGGGCAATTTGCCTCATTCTG |  |  |
| s640F | Pc21g12640 | TGTCTCTCTGTGGGCTGTTCTCAG |  |  |
| s640R | Pc21g12640 | CAAGAGTTCTTACGATGCGTGGCTG |  |  |
|  |  |  |  |  |
| abr1F | Pc21g16380 | GTCTACCTGAACTGGAACCTCACTTGG | **qPCR of DHN-melanin gene cluster** |  |
| abr1R | Pc21g16380 | GGTGAGTGTCAACTCTTCATCAAAGTGG |  |  |
| arp1F | Pc21g16420 | TCTTCAATTCTTCAGTCTCGTAGACCTGG |  |  |
| arp1R | Pc21g16420 | CCGAGCCCAACTTGGACATCAGC |  | **This study** |
| arp2F | Pc21g16430 | GAGGGTTTTACTCGCTGCTTTGCTGC |  |  |
| arp2R | Pc21g16430 | GATGTTAGCTCCACCGTTGCAAGGC |  |  |
| ayg1F | Pc21g16440 | GCTATGGCGGAGAAGTATGGCTATGAC |  |  |
| ayg1R | Pc21g16440 | CTCTCCAACCACTTGTAAGCCACAGG |  |  |
| abr2F | Pc22g08420 | GCTCAATGTAATGTCCATCCACCTCG |  |  |
| abr2R | Pc22g08420 | GACCCTGAGTATCTGACAAATCTCCAGC |  |  |
|  |  |  |  |  |
|  |  |  |  |  |

|  |  |  |  |
| --- | --- | --- | --- |
| attB4FΔHdaPF | Pc21g14570 | GGGGACAACTTTGTATAGAAAAGTTGCGTTTAAAGGCAGCCAAAGACTAAACTCAGTA | **Cloning/pHdaA/Sequencing/S.Blot** |
| attB1RΔHdaPR | Pc21g14570 | GGGGACTGCTTTTTTGTACAAACTTGCAAGGGAAAGCCACGGGAAGC |  |
| attB2FΔHdaPF | Pc21g14570 | GGGGACAGCTTTCTTGTACAAAGTGGCCTGATTCGAGCGTGAACCC |  |
| attB3RΔHdaPR | Pc21g14570 | GGGGACAACTTTGTATAATAAAGTTGTTTAAATGGTTGGTCACGACAGCGTT |  |
| ColonyPCR F1 | Pc21g14570 | CTCTTCACTGGCTTGTACATTCTGCG |  |
| ColonyPCR R1 | Pc21g14570 | ATTCACACGTGCTAGTGGAC |  |
| SeqΔHda1 | Pc21g14570 | AGGCAGCCAAAGACTAAACT |  |
| SeqΔHda2 | Pc21g14570 | GGAGGCAATAACTGCAGTAG |  |
| SeqΔHda3 | Pc21g14570 | GACGAGTGATGGTGATAGTTC |  |
| SeqΔHda4 | Pc21g14570 | GAACGTTGACATCGATCAC |  |
| SeqΔHda5 | AMDS cassette | GCGAGACAGTCAACAACATC |  |
| SeqΔHda6 | AMDS cassette | AGGAGCCATGGAAATACG |  |
| ProbeF | Pc21g14570 | GTACATCCATGGATGTTTCTGCTCATATTTGC |  |
| ProbeR | Pc21g14570 | CTTATATAGTTTCCCTGCTGGTGGATTGAGC |  |
|  |  |  |  |
|  |  |  |  |
| attB4FΔpks17 | Pc21g16000 | GGGGACAACTTTGTATAGAAAAGTTGGCTGTCATTGAGTCGCTAGGTTATCTCC | **Cloning / pKO17** |
| attB1RΔpks17 | Pc21g16000 | GGGGACTGCTTTTTTGTACAAACTTGCCAGTGGCGAATTATTGGTTTCAGGCG |  |
| attB2FΔpks17 | Pc21g16000 | GGGGACAGCTTTCTTGTACAAAGTGGGTGCCTACTTCCAGGACATTTGTATATGGG |  |
| attB3RΔpks17 | Pc21g16000 | GGGGACAACTTTGTATAATAAAGTTGGATTCAACTAACATTTGTGGCAGGACGAGG |  |
|  |  |  |  |
| attB4Foepks17 | Pc21g16000 | GGGGACAACTTTGTATAGAAAAGTTGGATGACCCACGTGCATAAGTGACAGC | **Cloning / pOE17** |
| attB1Roepks17 | Pc21g16000 | GGGGACTGCTTTTTTGTACAAACTTGCCAGTGGCGAATTATTGGTTTCAGGCG |  |
| attB2Foepks17 | Pc21g16000 | GGGGACAGCTTTCTTGTACAAAGTGGATGGAAGGCCCCGGTCATGTATATCTC |  |
| attB3Roepks17 | Pc21g16000 | GGGGACAACTTTGTATAATAAAGTTGCAAACATTCCGGCGTCGTTATACCAGC |  |

**This study**

**Figure S1.** A) Southern blot analysis of *P. chrysogenum* strains with individual *hdaA* gene deletions. B) Scheme of the replacement of the *hdaA* gene in *P. chrysogenum* with the amdS cassette. The length of DNA fragments detected by Southern Blot is indicated for *∆hdaA* and DS56530 strains. C) Scheme of PCR products sequenced from *∆hdaA* and DS56530 strains. Black arrows indicate the used primers (Colony PCR primers). Restriction enzyme used during the screening for positive colonies is marked.

**
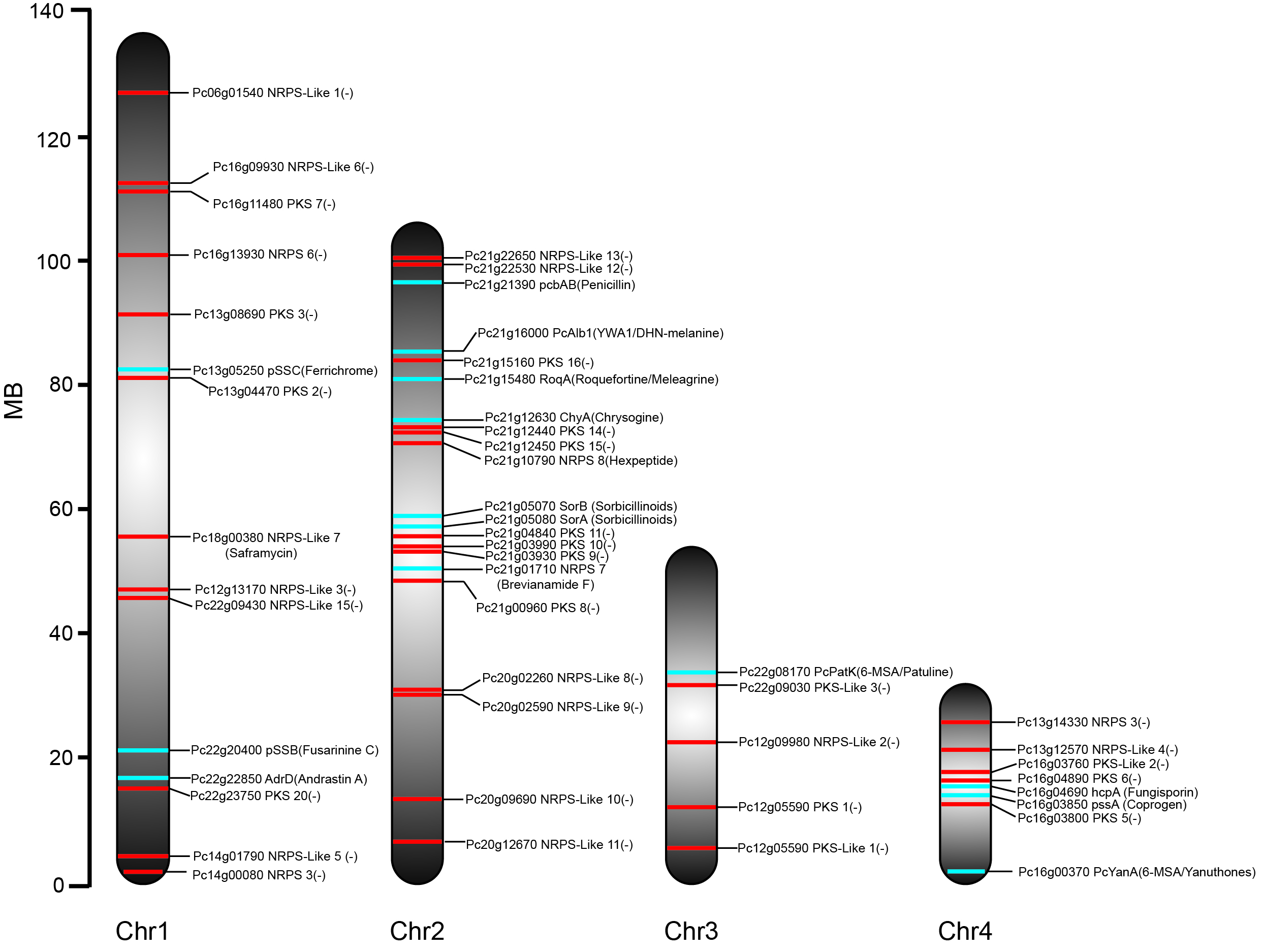
Figure S2.** Schematic representation and distribution of the (-like) PKSs and (-like) NRPSs genes over the four chromosomes of *P. chrysogenum*. *In silico* analysis were performed by comparison between gene number of *P. chrysogenum* Wis54-1255 and the homologue reported in industrial progenitor strain *P. chrysogenum* P2niaD18 (Specht *et al.*, 2014). Blue lines indicated known compounds. Red lines indicated unknown compounds. Adapted from (Ali *et al.*, 2013; Specht *et al.*, 2014; Salo *et al.*, 2016; Samol *et al.*, 2016; Guzman-Chavez *et al.*, 2017)

**Figure S3:** A) HPLC-MS extracted ion chromatogram (range m/z [M-H]^-^ 275.05-275.06) of the naphtho-γ-pyrone (YWA1) produced by *oepks17* strain (above) versus no production by *DS68530 (below)*. B) HPLC-MS spectra containing exact mass (m/z [M-H]^-^ 275.06) and calculated elemental formula (ppm 0.33) of the deprotonated naphtho-γ-pyrone (YWA1) together with the related fragment acquired by in-source (ESI) fragmentation in negative mode. Schematic representation of the fragmentation is shown.

**
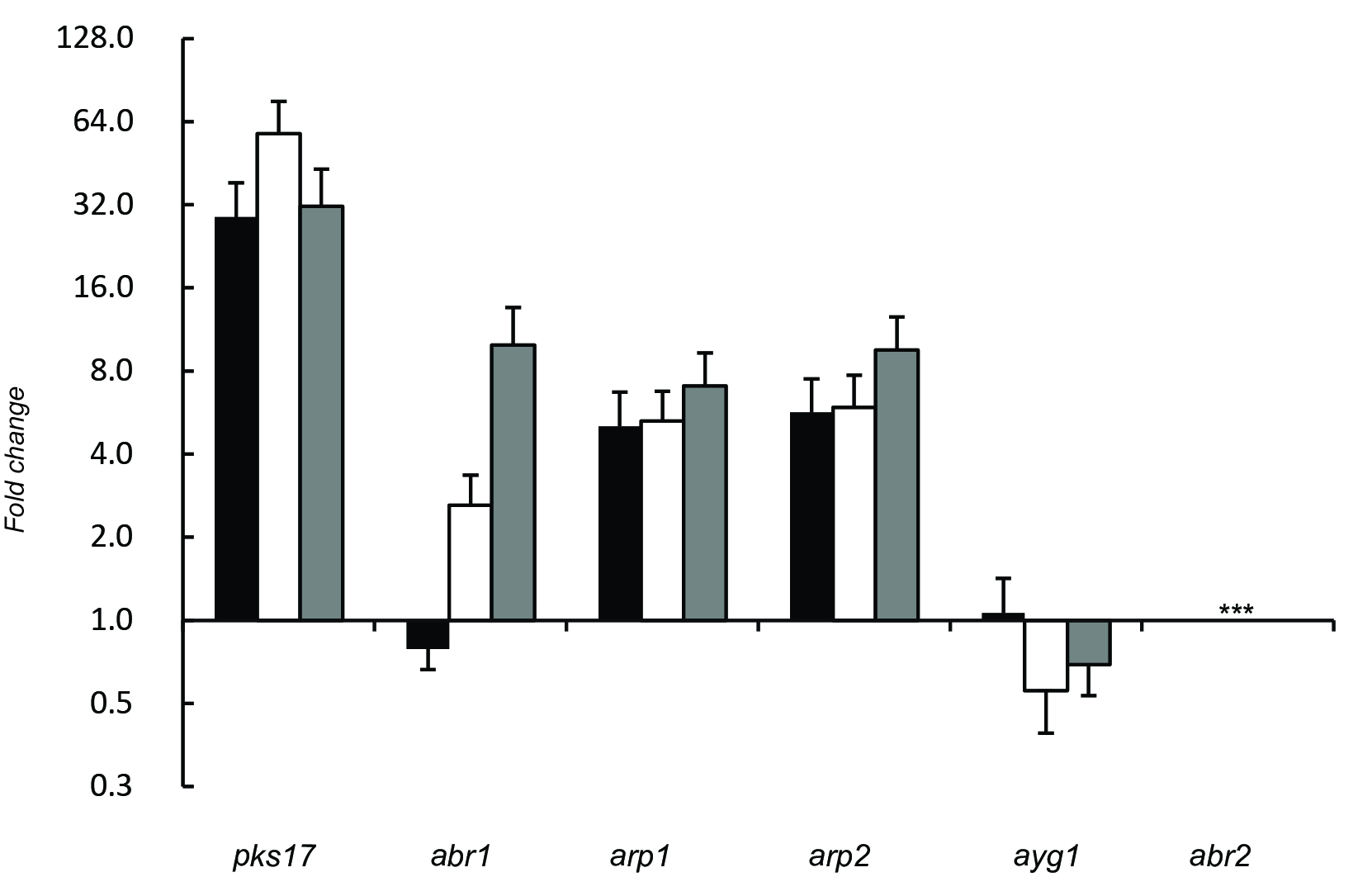
**

**Figure S4:** Quantitative Real Time PCR analysis of the gene cluster of DHN-melanin biosynthesis in SMP medium after 3 days of growth. Strains: DS685Res13 (black bars), ΔhdaA_DS68530Res13 (withe bars), and ΔhdaA_DS68530 (grey bars). Data are shown as a fold change relative to *P. chrysogenum* DS68530. (*∆hdaA*/DS68530). (*) Indicates non-detected expression under the tested strain. Error bars indicate the standard deviation of two biological with two technical replicates.

**References**

Ali, H., Ries, M.I., Nijland, J.G., Lankhorst, P.P., Hankemeier, T., Bovenberg, R.A., et al. (2013) A branched biosynthetic pathway is involved in production of roquefortine and related compounds in *Penicillium chrysogenum*. *PLoS One* **8**: e65328.

Guzman-Chavez, F., Salo, O., Nygard, Y., Lankhorst, P.P., Bovenberg, R.A.L., and Driessen, A.J.M. (2017) Mechanism and regulation of sorbicillin biosynthesis by *Penicillium chrysogenum*. *Microbial Biotechnology* **10**: 958–968.

Salo, O., Guzmán-Chávez, F., Ries, M.I., Lankhorst, P.P., Bovenberg, R.A.L., Vreeken, R.J., and Driessen, A.J.M. (2016) Identification of a Polyketide Synthase Involved in Sorbicillin Biosynthesis by *Penicillium chrysogenum*. *Applied and Enviromental Microbiology* **82**: 3971–3978.

Samol, M.M., Salo, O., Lankhorst, P., Bovenberg, R.A.L., and Driessen, A.J.M. (2016) Secondary Metabolite Formation by the Filamentous Fungus *Penicillium chrysogenum* in the Post-genomic Era. In, de Vries,R.P., Gelber,I.B., and Andersen,M.R. (eds), *Aspergillus and Penicillium in the Post-genomic Era*. Caister Academic Press, Norfolk, UK, pp. 145–172.

Specht, T., Dahlmann, T.A., Zadra, I., Kürnsteiner, H., and Kück, U. (2014) Complete Sequencing and Chromosome-Scale Genome Assembly of the Industrial Progenitor Strain P2niaD18 from the Penicillin Producer *Penicillium chrysogenum*. *GenomeA* **2**: 1–2.
